# Evaluation of fibrinolytic and antioxidant effects of *Allium elburzense* bulb extracts

**Published:** 2017

**Authors:** Leila Safaeian, Behzad Zolfaghari, Mohammadreza Aghaye-Ghazvini, Mahya Behnampour

**Affiliations:** 1 *Department of Pharmacology and Toxicology, Isfahan Pharmaceutical Sciences Research Center, School of Pharmacy and Pharmaceutical Sciences, Isfahan University of Medical Sciences, Isfahan* *, Iran*; 2 *Department of Pharmacognosy, School of Pharmacy and Pharmaceutical Sciences, Isfahan University of Medical Sciences, Isfahan* *, Iran*; 3 *Isfahan Center of Public Health Training and Research, Institute of Public Health Research, Tehran University of Medical Science, Iran*

**Keywords:** Allium elburzense Wendelbo, Antioxidant, Fibrinolytic agents

## Abstract

**Objective:**

*Allium elburzense* is an endemic plant of the family Amaryllidaceae that grows wild in northern Iran with some nutritional and medicinal applications. The present study was aimed to investigate the fibrinolytic and antioxidant effects of *A. elburzense *bulb extracts.

**Materials and Methods:**

Hydroalcoholic, aqueous, chloroformic and butanolic extracts were evaluated in this research. *In vitro *antioxidant assays were performed using total phenolic, DPPH, and FRAP methods. In the *in vivo* analysis, animals received i.p. injection of* A. elburzense* hydroalcoholic extract for 21 days and hydroperoxides level, FRAP value, PT and aPTT were determined in serum samples. The fibrinolytic activity of different extracts was quantitatively evaluated by measurement of clot weight.

**Results:**

*In vitro *antioxidant assay showed that *A. elburzense *aqueous extract had the highest DPPH scavenging and the highest total antioxidant capacity. In the *in vivo* assay, *A. elburzense* hydroalcoholic extract reduced serum hydroperoxides level and increased serum total antioxidant capacity in rats. *In vitro *fibrinolytic assay revealed remarkable thrombolytic activity for this plant with the highest effect for the aqueous extract. However, coagulation parameters including PT and aPTT were not affected by administration of *A. elburzense* hydroalcoholic extract in rats.

**Conclusion:**

In conclusion, the results of this study revealed the potential antioxidant and fibrinolytic effects of *A. elburzense* bulb extracts. For developing novel thrombolytic agents, further investigations for isolation of bioactive constituents and finding the underlying mechanisms are suggested.

## Introduction

Venous thromboembolism consisting of serious and life-threating complications such as deep vein thrombosis, post-thrombotic syndrome and pulmonary embolism, is of great clinical concern worldwide. This common preventable disorder contributes to long-term morbidity and high cost of health-care (Kesieme et al., 2011 ▶). Surgery, prolonged bed rest, congestive heart failure, pregnancy, nephrotic syndrome, cancer, obesity, chemotherapy and oral contraceptive therapy are some of important risk factors for venous thromboembolism (Anderson et al., 2003 ▶). 

In spite of different pharmacological treatments including antithrombotic and fibrinolytic drugs, venous thromboembolism remains a major cause of mortality and disability. Therefore, investigations for finding efficacious and safe antithrombotic drugs with low risk of bleeding are ongoing (Sikka et al., 2010 ▶). Recent studies have focused on herbal medicines as one of the potential sources of prevention and treatment of venous thromboembolism (Kim et al., 2015 ▶).

*Allium *is the major and most important genus of Amaryllidaceae family with more than 800 species in the world (Neshati et al., 2009 ▶). Some of important species such as onion, garlic and scallion have been used as foods, spices and medicine for a long time. *Allium* species have many biological effects and high potential for prevention and treatment of cardiovascular diseases including hypercholesterolemia, hypertension and atherosclerosis (Lister et al., 2007 ▶). Some species of this genus have been evaluated as possible anti-platelet-aggregation and antithrombotic agents (Srivastava et al., 1993 ▶; Hiyasat et al., 2009 ▶).

*Allium elburzense* Wendelbo is an endemic plant growing in the Alburz Mountains in northern Iran. This plant grows and blooms from April to May and its aerial parts are usually used as food. It has been traditionally used as an antidiabetic, antirheumatic, antihelminthic and aphrodisiac medicine. However, limited information has been reported on the pharmacological properties of *A. elburzense* (Zolfaghari et al., 2012 ▶). 

The present study was aimed to investigate the anticoagulant, fibrinolytic and antioxidant effects of *A. elburzense *bulb extract. 

## Materials and Methods

**Chemicals**

Streptokinase (SK) was purchased from Karma*-*Kinase Pharmatech GmbH (Germany). The standard assay kits for evaluation of 1,1-diphenyl-2-picrylhydrazyl (DPPH) radical scavenging, hydroperoxides concentration and ferric reducing antioxidant power (FRAP) assay were purchased from Hakiman Shargh Research Co. (Isfahan, Iran). Folin-Ciocalteu and all other reagents were purchased from Merck Co. (Mumbai, India).

**Plant material and preparation of extracts**

The bulbs of *A. elburzense *were collected from the Mount *Damavand* in the Alburz *Mountains*, Tehran province of Iran in March 2015. After identification of the plant by a Botanist (*Dr Iraj Mehregan*, Department of Biology, Science and Research Branch, Islamic *Azad University*, Tehran, Iran), a voucher specimen (No. 1145) was deposited at the Herbarium of the School of Pharmacy and Pharmaceutical Sciences, Isfahan, Iran. For preparation of hydroalcoholic extract, the air-dried bulbs of the plant (1200 g) were powdered and extracted with ethanol: water (70:30) using percolation method for 48 hr at room temperature. After filtration of the extract, the solvent was removed using a rotary evaporator and the extract was freeze-dried (Zolfaghari et al., 2015 ▶). 

For preparation of other extracts, dried ground bulbs (100 g) were sequentially extracted with hexane, chloroform, chloroform-methanol (9:1), water and butanol. After removal of saccharides, amino acids and solvents through aqueous solvent, the viscous residues of different extracts were obtained. All extracts were stored at -20 ºC (Zolfaghari et al., 2012 ▶). 

**Animals**

Male Wistar albino rats weighing 180 to 220 g were obtained from the animal house of the School of Pharmacy and Pharmaceutical Sciences, Isfahan, Iran. The animals had free access to water and standard rodent diet and were kept under standard laboratory condition with a 12 hr light/12 hr dark cycle. Rats were acclimatized for 1 week before the experiment. The experiment was conducted according to the international guidelines for laboratory animal use and care. For *in vivo* study, rats were randomly divided into 5 groups of 6 rats. Animals received daily intraperitoneal (i.p.) injections of *A. elburzense* hydroalcoholic extract (100, 200 and 400 mg/kg) or vitamin C (30 mg/kg, as a positive control) for 21 days (Zolfaghari et al., 2012 ▶; Kini et al., 2011 ▶; Onoja et al., 2014 ▶). Normal saline (i.p.) was used as the negative control. After the 21^st^ day, blood was collected through direct cardiac puncture under mild ether anesthesia and serum samples were used for further experiments.

***In vitro*** **antioxidant assay**

**Total** **phenolic assay **

Total phenolic content of the *A. elburzense *hydroalcoholic extract was determined using Folin-Ciocalteu reagent as previously described by Everette and coworkers (Everette et al., 2010 ▶). Results were obtained using a standard curve plotted based on different concentrations of gallic acid and expressed as milligram of gallic acid equivalent (GAE) /g of the dried plant extract.

**DPPH radical scavenging** **assay**

Free radical scavenging activity of all extracts was analyzed using 1,1-diphenyl-2-picrylhydrazyl (DPPH) radical scavenging method. Different concentrations of plant extracts (100 µl of 25-1000 µg/ml) were mixed with 100 µl of methanolic solution of DPPH (100 µM). The absorbance was measured at 517 nm after 30 min incubation in the dark at room temperature. The percentage of free radical inhibition was calculated using the formula:

 [(A_0_-A_1_)/A_0_] × 100

where A_0_ is the absorbance of the control (containing all reagents except the test compound), and A_1_ is the absorbance of the extract/standard. Ascorbic acid was used as the reference standard (Iwalewa et al., 2008 ▶). The half maximal inhibitory concentration (IC_50_) was calculated through a series of dose-response data and using an equation which was fitted to the curve. 

**FRAP assay**

The total antioxidant capacity of different plant extracts was determined by ferric reducing antioxidant power (FRAP) method. This assay is based on the reduction of ferric-tripyridyltriazine complex to ferrous form by colorimetric method. Briefly, the FRAP reagent (200 µl) containing tripyridyltriazine/ferric chloride/acetate buffer was freshly prepared according to the manufacturer’s protocol and added to 10 µl of the extracts (25-1000 µg/ml). The mixture was incubated for 40 min at 37 °C and then the absorbance of colored solutions was measured at 570 nm using a spectrophotometer (Bio-Tek, PowerWave XS, USA). The FRAP value of samples was calculated using a standard curve of FeSO_4. _7H_2_O and expressed as µM of ferrous sulfate equivalents per liter (Benzie et al., 1996 ▶).

***In vivo*** **antioxidant assay** **FOX1 assay**

The effect of hydroalcoholic extract of *A. elburzense* on serum hydroperoxides level was determined based on the ferrous ion oxidation by xylenol orange reagent (FOX1) method. Briefly, the FOX-1 reagent (200 µl) containing ammonium ferric sulfate was freshly prepared in aqueous medium with sorbitol according to the manufacturer’s protocol. Then, serum samples (10 µl) were mixed with the reagent and after incubation for 30 min at 37ºC, the absorbance of solutions was measured at 540 nm using a spectrophotometer (Bio-Tek, PowerWave XS, USA). The hydroperoxides concentration in serum samples was calculated using a standard curve of hydrogen peroxide (Wolf et al., 2014 ▶).

**FRAP assay**

The effect of hydroalcoholic extract of *A. elburzense* on serum total antioxidant capacity was evaluated by FRAP method as described previously in *in vitro* assay.

**PT and aPTT assay**

Prothrombin time (PT) and activated partial thromboplastin time (aPTT) were determined using plasma within two hours of sample collection by standard hematological methods as described by Dacie and Lewis (Dacie et al., 1995 ▶).

***In vitro*** **fibrinolytic assay**

The fibrinolytic activity was quantitatively evaluated by measurement of clot weight. Whole blood samples were collected from healthy human volunteers (*n *= 10) (aged 20-23 years) without a history of oral contraceptive or anticoagulant therapy after taking *informed* written *consent*. The blood specimen (400 µl) was moved to previously-weighed sterilized micro-centrifuge tubes and incubated at 37˚C for 30 min to form clots. After formation of clot, serum was completely removed without disturbing the clot and each tube was again weighed to determine the weight of the clot. For evaluation of thrombolytic activity, 100 μl of different plant extracts (5mg/ml) was added to micro-centrifuge tubes containing the clots. Streptokinase (SK; 30,000 I.U.; equivalent to its IC50) was used as a positive control and sterilized normal saline was considered as a negative non-thrombolytic control. After incubation of all tubes at 37ºC for 90 minutes, the released fluid was removed from the tubes and they were again weighed and the percentage of clot lysis was calculated (Ali et al., 2014 ▶).

**Statistical analysis**

Data were represented as the mean±SEM. For statistical significance, a one-way analysis of variance (ANOVA) followed by Tukey *post-hoc* test was used (SPSS software version 16.0). A P*<*0*.*05 was considered significant.

## Results

**Plant extracts**

The yield of the plant extract was 27.5 % (w/w) for hydroalcoholic, 3.15 % (w/w) for aqueous, 2.43 % (w/w) for chloroformic and 2.51 % (w/w) for butanolic extract.

***In vitro*** **antioxidant experiments**

**Total** **phenolic assay**

Evaluation of total phenolic content of the *A. elburzense *hydroalcoholic extract showed 33.52 ± 1.3 mg GAE/g of dried bulbs of the plant extract.

**DPPH radical scavenging** **assay**

**Figure 1:**
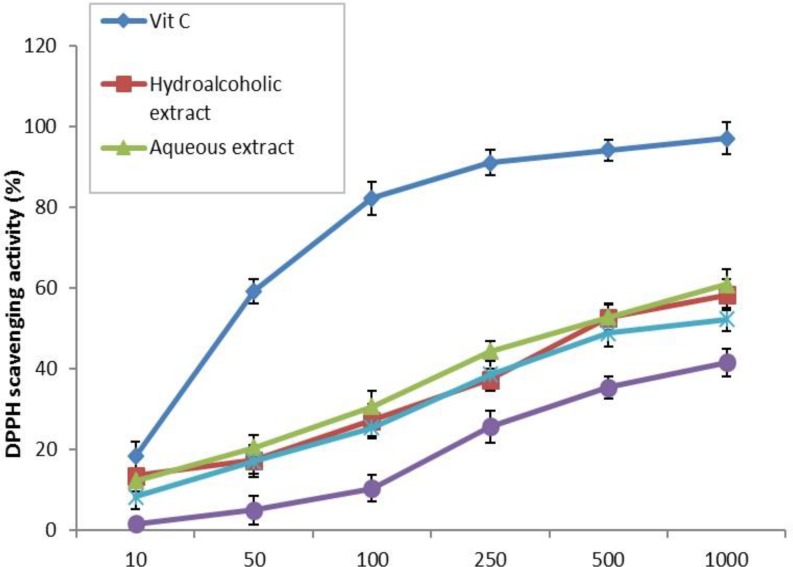
Scavenging activity of hydroalcoholic, aqueous, chloroformic and butanolic extracts of* A. elburzense *bulb and vitamin C (25-1000 µg/ml) against 1,1-diphenyl-2-picrylhydrazyl (DPPH). Results are means + SEM of three independent experiments

Antiradical activity of *A. elburzense* extracts was determined by DPPH scavenging test (Figure 1). IC_50_ of vitamin C as a standard antioxidant, was 0.034 mg/ml. The scavenging activity of different extracts was in the following order: aqueous extract (IC_50 _= 0.49 mg/ml) > hydroalcoholic extract (IC_50 _= 0.57 mg/ml) > chloroformic extract (IC_50 _= 0.94 mg/ml) > butanolic extract (IC_50 _= 1.68 mg/ml).

**FRAP assay**

**Figure 2:**
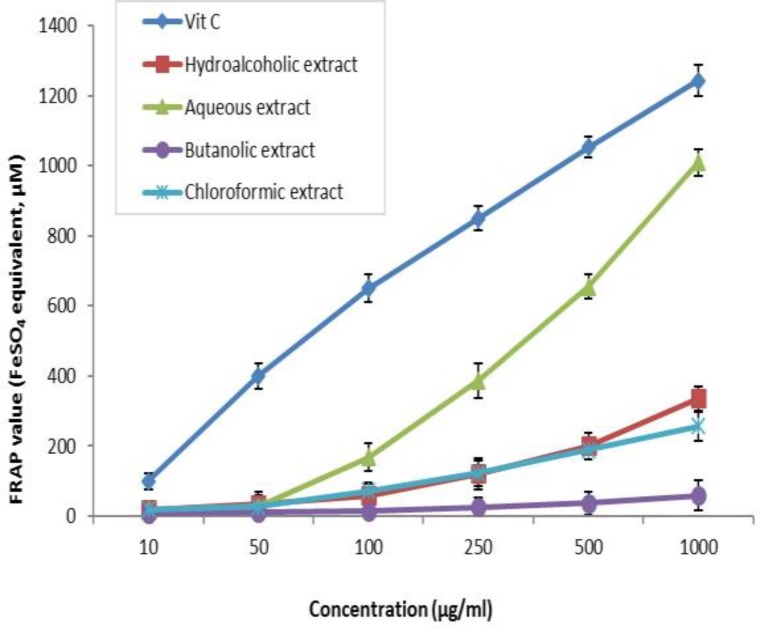
FRAP values of hydroalcoholic, aqueous, chloroformic and butanolic extracts of* A. elburzense* and vitamin C (25-1000 µg/ml) determined as ferrous sulfate equivalents. Values are means + SEM of three independent experiments

The total antioxidant capacity of different *A. elburzense* extracts was determined by ferric reducing antioxidant power (FRAP) method and expressed as the equivalents of ferrous sulfate. The extract showed a concentration-dependent increase in total antioxidant capacity (Figure 2). The antioxidant activity of plant extracts was in the following order: aqueous extract> hydroalcoholic extract ≈ chloroformic extract > butanolic extract.

***In vivo*** **antioxidant experiments**

**FOX1 assay**

**Figure 3:**
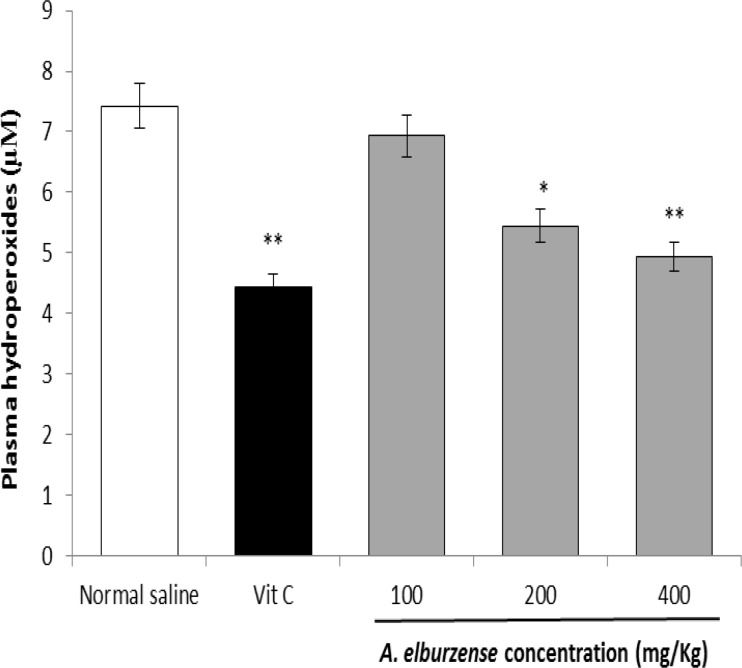
Effects of 21-day administration of *A. elburzense* hydroalcoholic extract (100-400 mg/kg) and vitamin C (30 mg/kg) on plasma hydroperoxides concentrations (as H_2_O_2_ equivalents) determined by FOX1 method. Values are means + SEM of six rats (^*^P<0.05, ^**^P<0.01 and^ ***^P<0.001 versus normal saline-treated control group

The effect of hydroalcoholic extract of *A. elburzense* on rats serum hydroperoxides level was determined using FOX1 Method. Administration of vitamin C as a reference standard, reduced serum hydroperoxides compared to the negative control rats (P<0.001). *A. elburzense* hydroalcoholic extract also significantly decreased serum hydroperoxides level at the doses of 200 and 400 mg/kg (P<0.5 and P<0.01, respectively) (Figure 3).

**FRAP assay**

**Figure 4:**
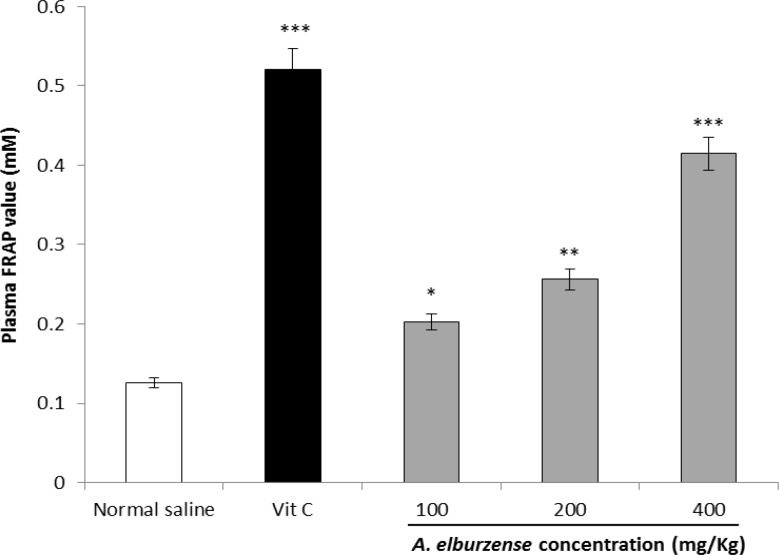
Effects of 21-day administration of *A. elburzense* hydroalcoholic extract (100-400 mg/kg) and vitamin C (30 mg/kg) on plasma FRAP value determined as ferrous sulfate equivalents. Values are means + SEM of six rats (^**^P<0.01 and^ ***^P<0.001 versus normal saline-treated control group

In the *in vivo* analysis, 21-day administration of vitamin C significantly elevated serum total antioxidant capacity compared to the control group (P<0.001). *A. elburzense* hydroalcoholic extract also increased the FRAP value dose-dependently (Figure 4).

**PT and aPTT assay**

**Figure 5:**
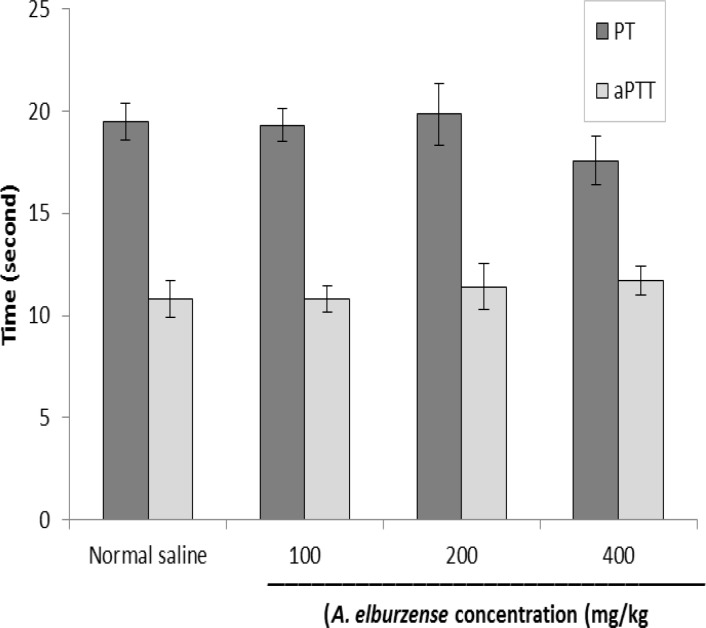
Effects of 21-day administration of *A. elburzense* hydroalcoholic extract (100-400 mg/kg) on coagulation parameters (PT and aPTT). Values are means + SEM of six rats

Administration of different doses of *A. elburzense* hydroalcoholic extract had no effect on coagulation factors PT and aPTT in the 21-day experiment (Figure 5). 

***In vitro*** **fibrinolytic assay**

**Figure 6:**
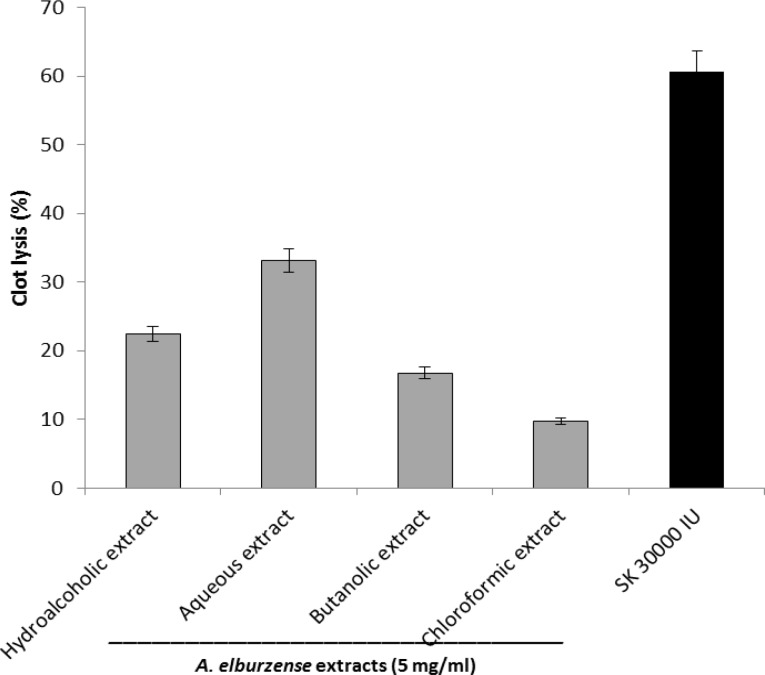
Thrombolytic activity of hydroalcoholic, aqueous, chloroformic and butanolic extracts of* A. elburzense* and streptokinase (SK; 30,000 I.U.) determined by measurement of clot weight. Values are means + SEM of three independent experiments

Streptokinase as a positive control (30,000 I.U.) caused 60.59% clot lysis after incubation for 90 min at 37°C. The thrombolytic activity of different extracts of the plant was in the following order: aqueous extract (33.11%)> hydroalcoholic extract (22.40%)> butanolic extract (16.75) > chloroformic (9.77%) extract (Figure 6).

## Discussion

Herbal medicine as a source of numerous bioactive compounds, is nowadays employed to alleviate various disease conditions (Babaee et al., 2016 ▶; Ghadirkhomi et al., 2016 ▶). *A. elburzense* is an endemic plant of northern Iran with nutritional and medicinal applications. However, pharmacological activities of this plant have not been completely studied (Zolfaghari et al., 2012 ▶). This study evaluated the anticoagulant, fibrinolytic and antioxidant effects of *A. elburzense *bulb extracts. 

*In vitro *antioxidant analysis of different extracts of the plant showed that *A. elburzense *aqueous extract had the highest DPPH scavenging and the highest total antioxidant capacity . In the *in vivo* analysis, *A. elburzense* hydroalcoholic extract reduced serum hydroperoxides level and increased serum total antioxidant capacity in rats. *In vitro *fibrinolytic assay also showed that *A. elburzense *aqueous extract had the highest thrombolytic activity . However, coagulation parameters including PT and aPTT were not affected by administration of *A. elburzense* hydroalcoholic extract in rats.

The antioxidant activities of various *Allium *spp have been reported. Garlic (*Allium sativum*) as a popular plant of Amaryllidaceae family, is rich in antioxidants. This plant has been widely investigated for prevention and treatment of many diseases associated with oxidative stress (Neil et al., 1994 ▶). Various antioxidant activities including ROS scavenging, increasing the cellular antioxidants (catalase, superoxide dismutase, glutathione peroxidase and glutathione), inhibition of lipid peroxidation and LDL oxidation, and protection of endothelial cells against oxidative damage have been reported for aged garlic extract (Borek et al., 2001 ▶). The antioxidant effects of *Allium *species may be attributed to different bioactive constituents including sulfur-containing compounds microelements, dietary fibers and polyphenols (Lanzotti., 2006 ▶; Gorinstein et al., 2005 ▶). The presence of flavonoids, phenols, steroids, glycosides and saponins has been reported in total extract of *A. elburzense *(Zolfaghari et al., 2012 ▶). In our study, the highest antioxidant effect of aqueous *A. elburzense *extract may be mainly due to the presence of phenolic and water-soluble organosulfur compounds in the aqueous soluble fraction.

*Allium *spp. especially garlic, also have valuable fibrinolytic and anti-platelet activities (Elsabban., 2009 ▶). Some bioactive components such as adenosine, allicin and thiosulfinates rgar are present in garlic are responsible for fibrinolysis effect, preventing platelet aggregation and inhibiting thromboxane formation (Ackerman et al., 2001 ▶). Steroid saponins from garlic and other *Allium *species are also involved in their anticoagulant effects (Matsuura et al., 2001 ▶). It has been reported that garlic does not change PT or PTT because it exerts its anticoagulant activity through interfering with platelet aggregation not with coagulation cascade (Fugh-Berman., 2000 ▶). Similarly, the results of our study showed no alteration in PT or PTT suggesting that the extrinsic and intrinsic pathways of coagulation were not affected by *A. elburzense *extract in rats.

The results of investigation for finding antithrombotic agents have led to the isolation of steroid saponins with fibrinolytic potential including proto-isoeruboside-B and isoeruboside-B from garlic (Matsuura et al., 2001 ▶). Yoshikawa and co-workers isolated two novel fibrinolytic saponins including lucyosides N and P from the seeds of *Luffa cylindrica* (Cucurbitaceae) (Yoshikawa et al., 1991 ▶). Anti-thrombotic activities have been reported for diosgenyl saponins extracted from the rhizome of *Dioscorea zingiberensis* through inhibition of factor VIII activities and platelet aggregation (Zhang et al., 2013 ▶). Several sapogenins and new saponins namely elburzensoides have also been isolated from the bulb of *A. elburzense *(Barlie et al., 2004 ▶; Barlie et al., 2005 ▶). 

Moreover, some other recognized compounds of *A. elburzense*, such as flavonoids and sulfuric compounds may be responsible for its fibrinolytic effect (Zolfaghari et al., 2012 ▶). Dar and his coworkers showed thrombolytic activity of a flavonoid compound through blocking an enzyme protein disulfide isomerase which was participated in blood clotting process (Dar et al., 2012). Some flavonoids isolated from the roots of *Scutellaria baicalensis* such as baicalein have shown fibrinolytic effect in cellular model (Kimura et al., 1997 ▶). Kaempferol is a flavonol with fibrinolytic potential which has been found in various plants including Allium *spp.* (Barlie et al., 2005 ▶, Rajput et al., 2011 ▶).

In conclusion, this study revealed the beneficial antioxidant and fibrinolytic effects of *A. elburzense* bulb extracts. Further investigations are needed for isolation of bioactive constituents responsible for thrombolytic activity of this plant and finding the underlying mechanisms and determining the therapeutic value of this herbal medicine. 
